# Perception and Quantization Model for Periodic Contour Modifications

**DOI:** 10.3390/jimaging8110311

**Published:** 2022-11-21

**Authors:** Dmitri Presnov, Andreas Kolb

**Affiliations:** Computer Graphics and Multimedia Systems Group, University of Siegen, 57076 Siegen, Germany

**Keywords:** glyphs, image-based visualization, contour modification, quantization model, perception model

## Abstract

Periodic, wave-like modifications of 2D shape contours are often applied to convey quantitative data via images. However, to the best of our knowledge, there has been no in-depth investigation of the perceptual uniformity and legibility of these kind of approaches. In this paper, we design and perform a user study to evaluate the perception of periodic contour modifications related to their geometry and colour. Based on the study results, we statistically derive a perceptual model, which demonstrates a mainly linear stimulus-to-perception relationship for geometric and colour amplitude and a close-to-quadratic relationship for the respective frequencies, with a rather negligible dependency on the waveform. Furthermore, analyzing the distribution of perceived magnitudes and the overlapping of the respective 50% confidence intervals, we extract distinguishable, visually equidistant quantization levels for each contour-related visual variable. Moreover, we give first insights into the perceptual dependency between amplitude and frequency, and propose a scheme for transferring our model to glyphs with different size, which preserves the distinguishability and the visual equidistance. This work is seen as a first step towards a comprehensive understanding of the perception of periodic contour modifications in image-based visualizations.

## 1. Introduction

**Figure 1:**
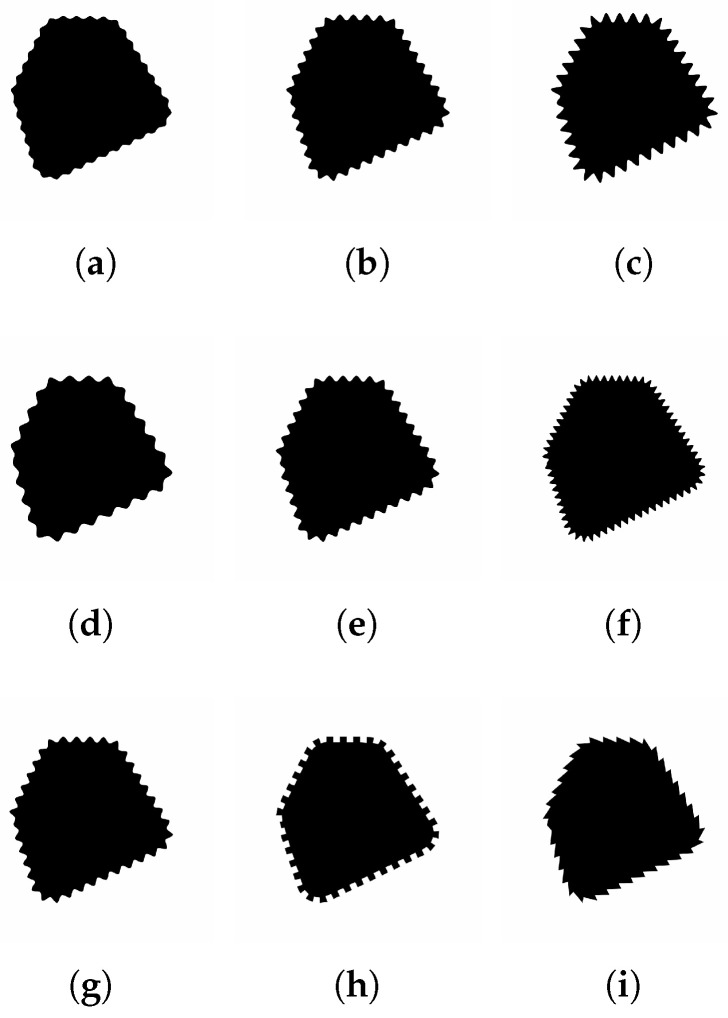
Examples of periodic contour modifications. (**a**–**c**): visual encoding by varying wave amplitude; (**d**–**f**): visual encoding by varying wave frequency; (**g**–**i**): visual encoding by varying waveform.

In visualization, the visual augmentation of contours is often used to extend the amount of available visual channels to represent further attributes of multidimensional data in parallel. This can be implemented, for instance, by variation of a contour’s *colour*, *width* and/or *fuzziness*, i.e., degree of blurring, or by means of a periodic, wave-like contour modification, which results in additional visual variables such as *frequency*, *amplitude*, and *waveform* (see Figure 1).

In Scientific Visualization, this kind of contour augmentation is applied, for example, to enhance the encoding capacity of isolines [1,2]. In Information Visualization, there are a number of approaches, which create glyphs or their parts from circular shapes, modulating their contours by a (mostly sinusoidal) wave. Such applications include the visualization of uncertainty (encoded by frequency [3,4] or amplitude [4]) and sport event data (encoded by frequency [5]) as well as the generation of more complex glyphs such as RoseShapes [6]. Besides circles, the contour waves have been also applied to iconic shapes such as leaf icons, to represent environmental data by means of frequency and amplitude [7].

Even if such contour waves prove to be a promising design solution, the perceptual aspects of the respective visual variables, however, have not been thoroughly investigated. While there are several studies that address the problem of perception and discrimination of more “classical” visual variables, e.g., colour [8,9], size [10], or the interdependencies of both [11], the work related to periodical contour modifications, to the best of our knowledge, is limited to the demonstration of the shapes’ orderability by frequency [3,12], based on user experiments, and to a rather abstract discussion about dependencies between wave amplitude and frequency without user studies [4].

Motivated by this fact, in this paper, we propose a *perception and quantization model* for periodic contour modifications, which provides a basis of glyph design for visualization of *multivariate quantitative* data. The proposed model addresses the essential properties of a quantitative visualization such as *perceptual uniformity* [13] and *accurate legibility* [14], performing a purposefully created user study and evaluating its results. *Perceptual uniformity* signifies that the representation of equally sampled data values have to be perceived in visual space as equidistant. However, mapping data to equally distributed discrete stimulus levels does not guarantee perceptual uniformity, since the relation between stimuli and sensation is generally not linear [15]. Therefore, our model comprises an estimation of an appropriate transformation function between stimulus magnitudes and perceived magnitudes. *Accurate legibility* corresponds to the distinguishability of the levels of a visual variable that encode quantitative data [14], which, in turn, implies a quantization, where the distances between single levels are equal or greater than the just noticeable difference (JND). To satisfy this requirement, we derived a quantization scheme, which aims to an optimal balance between the legibility and the visual capacity of the respective visual variable, i.e., the number of values it can encode [5].

**Figure 2:**
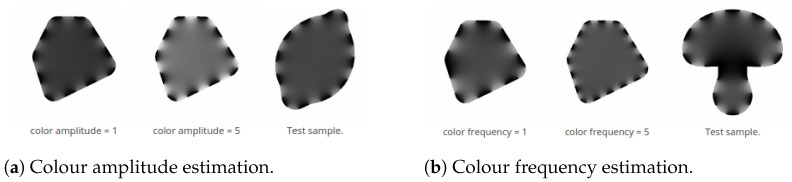
Design of different colour experiment types. (**a**,**b**) show examples for direct estimation of colour amplitude magnitude (with fixed colour frequency) and colour frequency magnitude (with fixed colour amplitude), respectively; the participants need to assess the corresponding magnitude in the “Test sample” (right) on the basis of the reference shapes (left and middle).

Since our goal is a generic approach that covers a large number of real world applications, we consider different waveforms and iconic shapes instead of a single, fixed geometric primitive such as a circle. Moreover, we take into account that the visual effect of contours modulated by a geometric wave can also be achieved by periodic modifications of the contours’ colour components, similarly to the “null-case glyph” in [3]. In particular, in this work, we focus on the colour modifications, created by alternation of the contours’ segments with different intensity levels (see Figure 2). Considering both, geometric modifications and colour modifications by varying intensity, we obtain a more generic model of periodic contour modifications that is evaluated in our user study.

To sum it up, our paper comprises the following contributions:

- An online user study about perception of periodic geometric and colour contour modifications.
- Modelling of a stimulus-to-perception transformation function for sinusoidal and colour contour modifications.
- Analysis of perceptual dependencies between amplitude and frequency for geometry and colour, respectively.
- Evaluation of the waveform influence on the amplitude and frequency perception, including a calibration model for sinusoidal, rectangular, and sawtooth waves.
- Definition of distinguishable quantization levels for geometric and colour contour modifications.
- A method for transferring the quantization model to shapes with different sizes.

## 2. Related Work

### 2.1. Periodical Contour Modifications in Visualization

There are several recent glyph visualization approaches that use periodical contour modifications, with application in different fields. For instance, Holliman et al. [3] used modified contours of a circular glyph, with wave frequency serving as a measure of visual entropy to encode uncertainty, while the inner colour of the circle visualizes the respective mean value. Similarly, Görtler et al. [4] proposed a contour-based design space for hierarchical uncertainty visualization by means of Bubble Treemaps, which includes, among possible alternative visual variables, sine wave frequency and amplitude as well as dashed frequency, whereby the latter can be considered as a kind of discontinuous rectangular wave. Here, mean values are encoded by circle size. Chung et al. [5] used contour wave frequency and radius of a circular silhouette as visual variables in a composite glyph for visualization of sport event analysis data.

Cai et al. in [6] followed a different approach, in which frequency, amplitude, and form of contour modifications do not serve as separable visual variables but as control parameters for construction of *unique* shapes, so-called RoseShapes, resulting from periodic functions plotted in polar coordinates.

On the contrary, in the glyph design for visualization of environmental data, developed by Fuchs et al. [7], the original leaf shapes maintain their recognizability and meaning after contour modifications, while frequency and amplitude of the resulting serrated boundaries can be used as additional visual channels.

### 2.2. Studies of Perception of the Contour Modifications

Since the visualization based on periodic contour modifications is a relatively new approach, its perception and discriminative capacity have not yet been investigated in detail and existing research is limited to contours of circular shapes. Besides psychophysical work that demonstrates the ability of the human visual system to discriminate shapes on the basis of radial frequencies (e.g., [16]), there are two recent visualization studies dedicated to the orderabilty issue. In particular, Chung et al. [12] investigated the suitability of specific visual channels to represent an ordinal scale. The results of their user study show that star shapes, which can be considered as circles modulated by a triangle wave with different frequencies, have an ordering. Furthermore, Holliman et al. [3] performed a user experiment to evaluate their uncertainty visualization approach and could demonstrate the orderability of circular glyphs with sinusoidal contours. Both studies used predefined frequency levels.

## 3. Materials and Methods

### 3.1. Components of the Perceptually Uniform Quantization Model

To develop a perceptually uniform quantization model of wave-like contour modifications, we evaluate the following aspects:

**Stimulus-to-perception transformation function,** i.e., transformation between stimulus magnitudes and perceived magnitudes. We assume that this function follows Stevens’s power law [15] and statistically estimates the corresponding parameters.
**Perceptual dependencies between amplitude and frequency.** Considering a pair of arbitrary geometric or colour amplitude and frequency values, the goal is to investigate how changes in one parameter influence the perception of the other.
**Perceptual influence of waveform** for geometric amplitude and frequency. It is assumed that the waveform of a geometric contour modification influences the perception of the respective amplitude and frequency. Thus, taken the sinusoidal shape as reference, the stimulus magnitudes for other shapes that produce the same sensation need to be acquired.
**Quantization** of visual variables, i.e., definition of clearly distinguishable and perceptually equidistant magnitude levels. We aim to achieve a balance between the number of available levels and their distinguishability.
**Size-dependent adaptation.** We propose rules for transferring the corresponding quantization to shapes with different sizes.

The expected model’s outcome are perceptually equidistant levels of each visual variable for data encoding, and their transformations to stimuli magnitudes and, where appropriate, to other geometric waveforms for glyph generation. The model is mainly derived from the results of an online user survey. However, it must be mentioned that not all aspects could be addressed equally in a single user study. First, the number of possible dependencies is in exponential relationship to the number of visual variables, and thus testing all of them in one study leads to an excessive experiment complexity and time exposure. Second, various advanced experiments imply a previous academic validation of primary test results, which are provided in this work.

Therefore, several investigation that are undoubtedly of high scientific interest could not be addressed in-depth in this first study. This mainly applies to the following: (1) The interferences between amplitude and frequency in geometry as well as in colour for which we, however, provide initial insight in Section 4.5. (2) The experimental validation of our rules to transfer our model to shapes of different sizes (Section 5). (3) The dependencies resulting from a combination of geometric and colour modifications. All these aspects have to be addressed in future work.

### 3.2. Design of the Experiment

**Figure 3:**
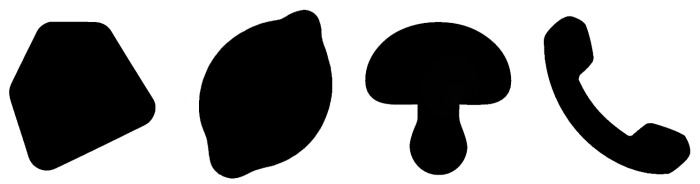
The four base shapes used for generation of modified contours in the experiments.

**Figure 4:**
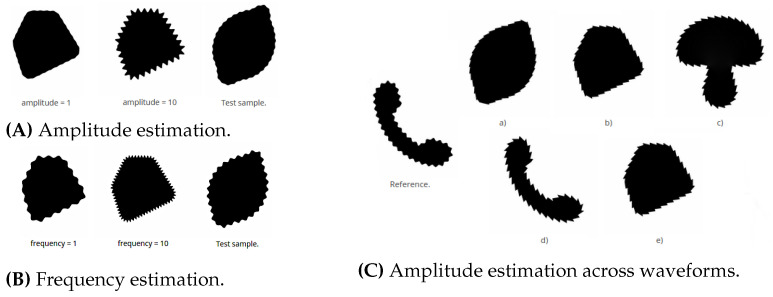
Design of different geometric experiment types. (**A**,**B**) are examples for direct estimation of amplitude (with fixed frequency) and frequency (with fixed amplitude) magnitude, respectively; the participants need to assess the corresponding magnitude in the “Test sample” (right) on the basis of the reference shapes (left and middle). (**C**) shows an example of a sawtooth amplitude calibration against a sinusoidal reference; the task is to select the test shape (**a**–**e**) whose amplitude is perceived as the closest to the “Reference” shape (left).

The test samples used in the survey are created from four monochrome base shapes with white background and black foreground (see Figure 3). The geometric modifications are produced by modulation of the shape contours according to the given *geometric frequency, amplitude, and waveform*, namely sinusoidal, rectangular, and sawtooth-like (see Figure 1 and Figure 4). We accordingly narrow the amplitude and frequency range used in our experiments, as strong perceptual interferences are to be expected for extremely low and extremely high magnitudes (see [4]).

The intensity modifications result from alternating intervals of given length (i.e., inverse *colour frequency*) along the contour (see Figure 2). The intensity inside each next interval changes between the current modified value (i.e., *colour amplitude*) and the original black foreground (i.e., colour amplitude =0). Since all images used in the experiments have white background, the maximal colour amplitude is limited to a value resulting in a light grey colour, in order to maintain contrast.

All shape images have size 512×512 px and are displayed at size 50×50 mm. Table 1 summarizes the metric values used to generate the stimuli and gives the mapping to the stimulus parameter values used for communication in the experiment (see also Figure 4A,B). In each question, the base shape was selected randomly. The survey comprises two main categories of experiments.

**Magnitude estimation.** We performed several magnitude estimation experiments [15] to determine a proper quantization of the visual variables as well as the transformation function between the stimuli and perception parameters.
  For each visual variable to estimate, the participants got displayed the available magnitude range by presenting a minimum and maximum reference shape with the corresponding stimulus parameter values in arbitrary digital unit (adu; for mapping of the metric or intensity values to the respective adu, see Table 1). Figure 4A,B shows the design of the magnitude estimation experiments for geometry, and Figure 2a,b for colour. The test shape with randomly selected magnitude was hidden by default and was uncovered for eight seconds by clicking the corresponding button, and the participants had to assign the perceived magnitude from a drop-down list (the respective magnitude ranges available for selection are displayed in Table 2). The stepsize for generating the visual stimuli for the test shapes (see Table 1) was selected to be below a conservatively estimated JND, i.e., significantly smaller than the distance distinguishable by the experiment designers, to be able to derive a suitable quantization from a statistical evaluation.
  There are two subtypes of the magnitude estimation experiments in our survey (see Table 2):
  1. Fixed second stimulus, e.g., geometric amplitude estimation with a fixed geometric frequency.
  2. Randomly selected second stimulus.
  The experiments with fixed second stimulus had been placed at the beginning of the specific experiment section to make the participants acquainted with the experimental setting, as the experiments with randomly selected second stimulus are more challenging.
**Waveform-dependent calibration** has been performed by selecting the modified shapes with the closest magnitude. To reduce the number of questions, all magnitude estimation experiments for geometric visual variables are done with the sinusoidal waveform. To estimate a waveform calibration function, the participants had to select one out of five glyphs with the perceptually most similar magnitude to a presented sinusoidal reference (see Figure 4C). The modified shapes offered for selection were created with the magnitude levels $l\in [l^{\mathrm{ref}}-2.\;.l^{\mathrm{ref}}+2]$, where $l^{\mathrm{ref}}$ denotes the visual variable values (adu) used for the reference shape, and have been arranged randomly. These experiments were done separately for each waveform, i.e., rectangular or sawtooth-like, and for each visual variable (see Table 2).
  We additionally performed one experiment to verify the visual distinguishability between the three waveform types—sinusoidal, rectangular and sawtooth-like—for combinations of low frequencies/low amplitudes and high frequencies/high amplitudes not listed in Table 2. The recognition rates were approximately 92%, 99%, and 99% for sinusoidal, rectangular, and sawtooth-like, respectively.

The design of the experiment assumes to have “cooperative” participants, i.e., participants that will not “cheap their way through” the experiment, and that the time limit for the ability to concentrate is at most 20–25 min. Table 2 states the number of experiments taken per experiment type. Each participant was asked to go through 90 experiments in total.

### 3.3. Survey Evaluation

We invited students and researchers mainly from our university from the fields of computer science and sociology to participate in our online survey, and an anonymous group of 73 persons participated. The average time to take the survey was ≈26 min. Given the raw results from the survey experiments conducted by the participants, we determined the required stimulus-to-perception transformation, quantization, and calibration parameters after having applied an outlier removal.

#### 3.3.1. Outlier Removal

First, the “senseless” answers are filtered out, i.e., answers which deviate from the expected value to an extend not explainable by the subjective character of perception alone. These outliers are mainly caused, e.g., by a misunderstanding of the respective experimental setting or by an external distraction of the participant while conducting the experiment. We apply the two-step Chebyshev outlier detection method of Amidan et al. [17], with the filtering parameters $p_{1}=0.375$ and $p_{2}=0.175$ for all visual variables.

#### 3.3.2. Modelling the Stimulus-to-Perception Transformation Function

Following Stevens [15], we assume that the stimulus-to-perception transformation has the form of a power function $e\left(x\right)=a\cdot x^{b}+c$. Thus, having the perceived magnitudes, as stated by the participants, as data points e(x) and the stimulus magnitudes x as the independent parameter, *a*, *b*, and *c* are estimated using nonlinear least-square fitting.

#### 3.3.3. Quantization

The aim is to find a quantization step Δv in perceptual space such that all resulting magnitude levels do not overlap with neighbouring confidence intervals for a given confidence level. This is analogous to the principle applied by estimation of just noticeable difference (JND), which is also defined regarding the probability of correct assignments, usually 50%, which we also apply in our experiment. Table 3 gives an overview of the quantization steps and the resulting number of discrete levels for each visual variable.

More precisely, the quantization step is calculated as follows:

1. For each discrete stimulus magnitude level, observe and model the distribution of perceived magnitudes.
2. Compute the 50% confidence interval, symmetrically placed about the respective mean.
3. Use the largest confidence interval as Δv.

#### 3.3.4. Waveform-Dependent Calibration

We aim to define a function that calibrates the stimulus amplitude and frequency of a rectangular or sawtooth-like waveform against the respective parameters of a sinusoidal shape. This calibration is modelled as a linear function h(x)=k·x+l, with x being the reference sinusoidal magnitudes from the calibration experiments (see Section 3.2) and the perceived magnitudes regarding the rectangular or sawtooth-like waveform as data points. The fitting of *h* is done with a least-square method.

#### 3.3.5. Evaluation of Perceptual Dependencies between Amplitude and Frequency

To evaluate the perceptual dependencies between geometric amplitude and frequency, we additionally estimate stimulus-to-perception transformation functions of the investigated visual variable $v_{1}$ in dependence on the magnitude of the respective second visual variable $v_{2}$. To get a sufficient number of samples and taking advantage of the fact that the differences between neighbouring magnitudes are sufficiently small, we pool $v_{2}$ in intervals. The resulting extended transformation functions for amplitude and frequency, respectively, are

$$\begin{array}{cc} \cdot a_{p} & =e(\cdot a_{s},\left\{\cdot f_{s}\right\}),\\ \cdot f_{p} & =e(\cdot f_{s},\left\{\cdot a_{s}\right\}), \end{array}$$

where · is a placeholder for *g* (geometry) and *c* (colour), and the second function parameter is a set of adu levels of $v_{2}$, which serves as a mask, i.e., only samples where $v_{2}$ has a corresponding value are considered. For instance, $e(ga_{s};\left\{2,3,4\right\})$ gives perception of stimulus geometric amplitude $ga_{s}$ with geometric frequency $gf_{s}\in \left\{2,3,4\right\}$. The function fitting is performed in the same way as described in Section 3.3.2.

For geometry, we pool $v_{2}$ in three intervals of three levels each. Since colour visual variables have been tested with five adu levels in the user study, we apply the following weighted pooling scheme to get three intervals again. Exemplarily, we pool the colour frequencies as

$$e\left(ca_{s},\left\{1,1,2\right\}\right),\;e\left(ca_{s},\left\{2,3,3,4\right\}\right),\;e\left(ca_{s},\left\{4,5,5\right\}\right),$$

where a double occurrence of a level in a set signifies that it is considered twice, i.e., weighted with factor 2. Colour amplitudes are pooled analogously.

## 4. Results

### 4.1. Outlier Removal

**Figure 5:**
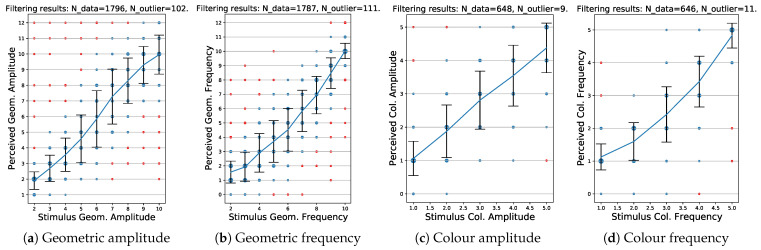
Results of the two-step Chebyshev outlier detection: outliers are marked in red; the point size encodes the number of occurrences.

Table 4 gives an overview for all visual variables. The overall amount of removed outliers is 4.8%. The detailed statistics of the outlier removal are represented in Figure 5.

### 4.2. Stimulus-to-Perception Transformation Function

**Figure 6:**
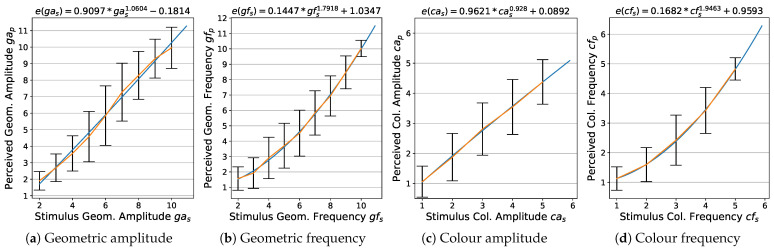
Modelling stimulus-to-perception transformation: blue: fitted transformation functions; orange: lines connecting perceptual means.

The modelled functions for transformation of stimuli into perceived magnitudes are presented in Figure 6 (for the estimation method, see Section 3.3.2). For both, geometry and colour amplitude, we observe a mainly linear and positive power dependency of the perceived magnitudes on the stimulus magnitudes, with the exponent b=1.0604 and b=0.928, respectively. The corresponding relationship for frequency is in both cases close to quadratic, b=1.7918 and b=1.9463.

### 4.3. Quantization

**Figure 7:**
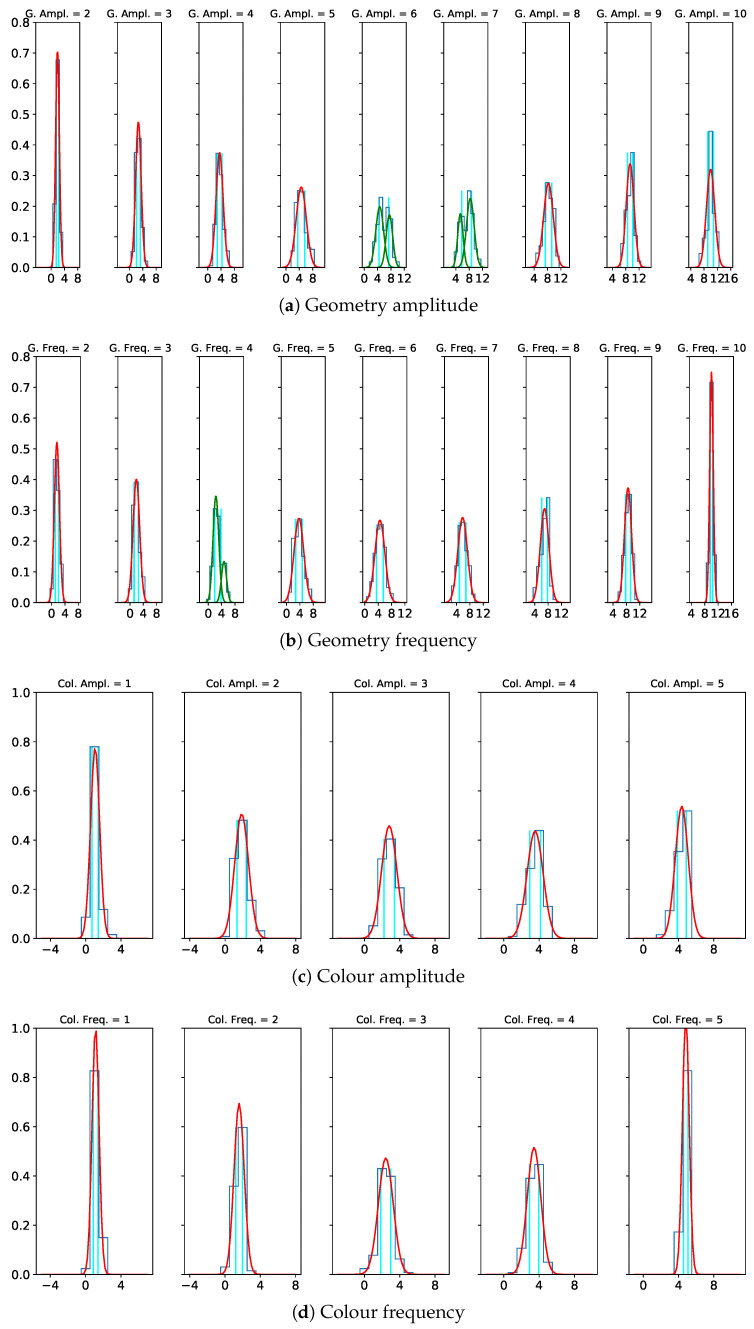
Perceived magnitudes as normal distributions. Red: simple Gaussian; green: two-component Gaussian mixture; cyan: borders of 50% confidence intervals.

The distributions of the perceived magnitudes reveal a mono-modal Gaussian nature for most magnitude levels and a bi-modal Gaussian behaviour for medium values of the geometric amplitude and frequency. The latter can be explained by a larger distance to the min. and max. references, which can be seen as a design-related phenomenon. Figure 7 shows the distribution of the perceived magnitudes with the respective 50% confidence intervals for each visual variable. We compute the 50% confidence for a bi-modal Gaussian distribution by identifying the 25% and 75% limits of the cumulative distribution of the superposition of both Gaussians. For the calculation methods, see also Section 3.3.3.

Table 3 gives an overview of the quantization steps and the resulting number of discrete levels for each visual variable. We observe that the distinguishability of both geometric and colour frequency is slightly better, i.e., their quantization steps are smaller than in the case of the respective amplitudes.

### 4.4. Waveform Calibration

**Figure 8:**
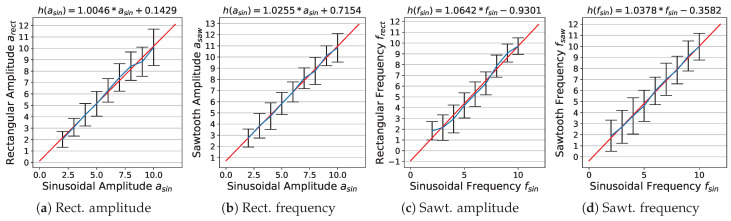
Calibration of rectangular and sawtooth-like waveforms against sinusoidal waveform: red: fitted calibration functions; blue: lines connecting perceptual means.

Figure 8 shows the modelled linear calibration functions (for the estimation method, see Section 3.3.4). The data demonstrate that the influence of a specific waveform on the perception of amplitude and frequency magnitudes is rather marginal, i.e., k≈1 for all four parameters.

### 4.5. Evaluation of Perceptual Dependencies between Geometric Amplitude and Frequency

**Figure 9:**
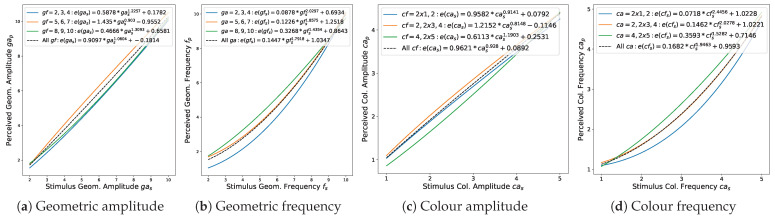
Modelling stimulus-to-perception transformation in dependence on the magnitude of the second visual variable.

The modelled functions for transformation of stimuli to perceived magnitudes in dependence on the second visual variable are presented in Figure 9 (see Section 3.3.5 for the evaluation method). For the geometric amplitude (Figure 9a), we observe slightly higher perceived magnitudes for medium frequencies, which corresponds to an approximately linear stimulus–perception relationship with the exponent $b_{medium}=0.903$ (see Section 4.2), while for low and high frequencies, the transformation function is close to the square root form with $b_{low}=1.2257$ and $b_{high}=1.3093$, respectively. For the geometric frequency (Figure 9b), the perceived magnitudes increase with increasing amplitudes, i.e., the transformation function varies from a weaker to a more pronounced exponential function with $b_{low}=1.4345<b_{medium}=1.8575<b_{high}=2.0297$. A similar trend can be observed for the colour frequency (Figure 9d). However, in this case, it can be explained by the contrast at the colour interval borders: while high colour amplitudes lead to hard transitions, making the interval alternation more salient, low amplitudes produce a kind of blurry borders, making the intervals seem larger. Finally, colour amplitude perception does not show any apparent pattern in its dependency on colour frequency (Figure 9c). In general, for all four visual variables, the respective deviations are rather marginal.

## 5. Transfer to Different Shape Sizes

The quantization levels of visual variables derived in Section 4 are based on the experiments with a fixed image size 50×50 mm. To allow a flexible application in different visualization contexts, we propose a scheme of how to apply our quantization results to glyphs with different sizes, even though the evaluation of this scheme must be deferred for future research. Note that our scheme does *not generate visual variables that are comparable across different scales*.

Before starting with the definition of the transferring rules, first, we sum up the quantization process for the fixed size, introducing some necessary notation. More precisely, for a perceived visual variable *v*, we initially fixed the minimum and the maximum stimulus values $s_{\min},s_{\max}$ (in mm) and deduced the quantization size Δv (in adu) applied to the range $v_{\min},v_{\max}$ (in adu) from the user experiment, which corresponds to $s_{\min}$ and $s_{\max}$ (see Section 3.3). Note that Δv corresponds to Δs(v) (in mm), which is in general not constant (see Figure 6).

To transfer the aforementioned quantization parameters to shapes with a relative scale ω>0 to the original shape of 50×50 mm, we propose the following rules, where we assume 0<ω<1, since glyphs are rather used at smaller scales:

- Colour amplitude should not be scaled, as intensity is independent of size.
- The “perceptual” stepsize Δv (and the corresponding stimulus stepsizes Δs(v)) should not be reduced to preserve the absolute variation (in mm), and thus, the visual distinguishability.
- The minimum and the maximum stimulus and visual variable values $s_{\min},s_{\max}$ and $v_{\min},v_{\max}$, respectively, are scaled according to the following rules:
  − The minimum values $s_{\min}$ and $v_{\min}$ can only be scaled moderately, i.e., reduced using $\omega_{\min}>\omega$, potentially even $\omega_{\min}=1$, to prevent, for example, visually vanishing amplitudes.
  − The maximum values $s_{\max}$ and $v_{\max}$ should be scaled by $\omega_{\max}=\omega$, to prevent, for example, extreme distortions for small shapes.
  Consequently, the number of levels gets potentially reduced for ω<1 as the “usable” range $\left[\omega_{\min}\cdot s_{\min},\omega_{\max}\cdot s_{\max}\right]$ gets smaller while the stepsize Δs(v) remains unchanged. To counteract on this problem, we propose to reduce the scaling effect for the maximum values $\omega_{\max}=\omega +\epsilon \leq 1$ with a user-defined parameter ϵ that also depends on the shape’s complexity.

**Figure 10:**
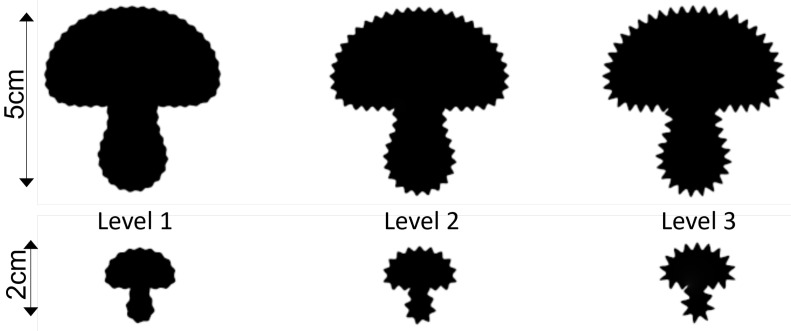
Transfer of quantization results to different shape’s sizes: geometric amplitude levels $l_{1},\ldots ,\;l_{3}$. Top row: original size. Bottom row: scaled with ω=0.4. This figure needs to be displayed according to the given scale. Note that the quantization levels are *not intended to be comparable across scales*.

Figure 10 shows some exemplary results of the quantization transfer to shapes with ω=0.4.

## 6. Conclusions, Limitations, and Future Work

### 6.1. Summary

In this paper, based on the results of a user study, we defined a perceptually uniform quantization model of periodical contour modifications for a glyph-based visualization design, comprising the visual variables such as geometric amplitude and frequency, waveform as well as colour amplitude and frequency. The main model components are stimulus-to-perception transformation, waveform-dependent calibration, and definition of the quantified levels for the corresponding visual channels. Moreover, we evaluated the potential impact of the perceptual dependencies between specific visual variables.

Below, we first sum up our core findings:

1. Following [15], the *relation between stimuli and their perception* for all four quantitative visual variables, considered in the model, can be modelled as a power function (see Section 4.2). Since the adu-scale used in the experiments does not have a proper zero-origin, we extended the power function with an additive term to compensate this fact.
2. The *influence of waveform* on the perception of geometric amplitude and frequency is marginal (see Section 4.4). As a consequence, the corresponding calibration step in a visualization design can be skipped.
3. The user study shows that the geometric as well as the colour frequency have a better *discriminative capacity* than the respective amplitudes (see Section 4.3). Overall, the geometric frequency has the highest number of quantified levels in the tested range.

### 6.2. Limitations

Additionally to these results, our study also allows the following assumptions regarding further perceptual aspects of the contour modifications, which still require an in-depth investigation or validation in future work:

1. A first insight into *amplitude–frequency dependencies*, provided in this work, shows certain perceptual trends as a function of the respective second parameter, but the resulting deviations are rather marginal and thus can be likely neglected by a visualization design (see Section 4.5).
2. We propose a method to transfer our model, estimated for shapes with a fixed size 50×50 mm, to *arbitrary sizes* (see Section 5). We heuristically derived the respective rules and showed first exemplary results created with this method.

### 6.3. Future Work

Finally, our results can serve as inspiration for some related topics, which, however, are beyond the scope of this study:

1. The current quantization has been statistically estimated on the basis of perceptual data. It may be of interest to compare our results with other estimation methods, for instance, direct JND tests.
2. We consciously narrowed the corresponding ranges of the geometric visual variables to avoid the expected strong interferences for low and high magnitudes [4], as mentioned in Section 3.2. At the same time, we assume that the current maximum is still relatively far away from critical magnitudes. Consequently, the current *limit* needs further investigation in a separate experiment. Furthermore, we assume that the *amplitudes and frequency limits* depend to some degree on the respective base shape, especially on its local curvature.
3. Colour contour modifications, limited in the current user study to black-white images, can be transferred to shapes with *other foreground colours*, but a potential reduction of the number of colour amplitude levels, depending on the base shape intensity and the resulting shift of zero amplitude, has to be taken into account.
4. A *combination of* two main modification types—*geometry and colour*—is also conceivable. According to a specific visualization design, it can be implemented as four independent quantitative visual variables as well as in a coupled form, e.g., with colour frequency equal to geometric frequency and colour amplitude linked to geometric amplitude. Such combinations potentially entail dependencies between colour and geometry perception.

## Figures and Tables

**Table 1:** Metric values for the experiment with the glyph size 50 mm. The number in {}-brackets are the corresponding visual variable values (arbitrary digital unit, adu) used for communication in the experiment. Note that the frequency is proportional to the inverse period length. The colour amplitude values are given as value/brightness V in [0,1], in HSV colour space.

Visual Variable	min [mm]	max [mm]	step [mm]
Geometric amplitude	0.1 {1}	1.2 {12}	0.1
Geometric period length	0.8 {12}	5.1 {1}	0.4
Colour period length	5.9 {5}	12.1 {1}	1.6
	**min [V]**	**max [V]**	**step [V]**
Colour amplitude	0.425 {1}	0.85 {5}	0.10625

**Table 2:** Overview of survey experiments. Each row is one experiment type, where the visual variables, i.e., the perceptional parameters to be assessed, are plotted **bold-faced**. Other parameters might be fixed values, indicated by *F*, or randomly selected, indicated as *R*. The numbers given in []-brackets are the stimuli values defined in Table 1. The individual experiments are of two kinds: $\left[2.\;.10\right]\to \left[0.\;.12\right]$, for example, generates stimuli in the value range $\left[2.\;.10\right]$ and asks for assessing the perceptional values in $\left[0.\;.12\right]$, while $x_{|\in \left[2.\;.10\right]}\to \left[x-2.\;.x+2\right]$ generates stimuli values *x* in the range $\left[2.\;.10\right]$ and asks for assessing the perceptional values in the dependent range $\left[x-2.\;.x+2\right]$.

Experim.	Waveform	Geometric Amplitude	Geometric Frequency	Colour Amplitude	Colour Frequency	# exp.
**Ampl1**	sin.	$\left[2.\;.10\right]\to \left[0.\;.12\right]$	$F\left[6\right]$	n.a.	n.a.	6
**Ampl2**	sin.	$\left[2.\;.10\right]\to \left[0.\;.12\right]$	$R\left[2.\;.10\right]$	n.a.	n.a.	20
**Freq1**	sin.	$F\left[6\right]$	$\left[2.\;.10\right]\to \left[0.\;.12\right]$	n.a.	n.a.	6
**Freq2**	sin.	$R\left[2.\;.10\right]$	$\left[2.\;.10\right]\to \left[0.\;.12\right]$	n.a.	n.a.	20
**SawtAmpl**	sin.→sawt.	$x_{|\in \left[2.\;.10\right]}\to \left[x-\mathrm{method}2.\;.x+2\right]$	$F\left[6\right]$	n.a.	n.a.	5
**RectAmpl**	sin.→rect.	$x_{|\in \left[2.\;.10\right]}\to \left[x-2.\;.x+2\right]$	$F\left[6\right]$	n.a.	n.a.	5
**SawtFreq**	sin.→sawt.	$F\left[6\right]$	$x_{|\in \left[2.\;.10\right]}\to \left[x-2.\;.x+2\right]$	n.a.	n.a.	5
**RectFreq**	sin.→rect.	$F\left[6\right]$	$x_{|\in \left[2.\;.10\right]}\to \left[x-2.\;.x+2\right]$	n.a.	n.a.	5
**ColAmpl1**	n.a.	n.a.	n.a.	$\left[1.\;.5\right]\to \left[0.\;.5\right]$	$F\left[3\right]$	3
**ColAmpl2**	n.a.	n.a.	n.a.	$\left[1.\;.5\right]\to \left[0.\;.5\right]$	$R\left[1.\;.5\right]$	6
**ColFreq1**	n.a.	n.a.	n.a.	$F\left[3\right]$	$\left[1.\;.5\right]\to \left[0.\;.5\right]$	3
**ColFreq2**	n.a.	n.a.	n.a.	$R\left[1.\;.5\right]$	$\left[1.\;.5\right]\to \left[0.\;.5\right]$	6

**Table 3:** Quantization results derived from the user experiment with the image size 50×50 mm.

Visual Variable	Geometric Amplitude	Geometric Frequency	Colour Amplitude	Colour Frequency
Quant. step Δv (adu)	≈2.91	≈2.01	≈1.23	≈1.14
# levels (50 mm)	4	5	4	4

**Table 4:** Outlier removal results.

Visual Variable	Geometric Amplitude	Geometric Frequency	Colour Amplitude	Colour Frequency
**# data**	1796	1787	648	646
**# outliers**	102	111	9	11

## Data Availability

Not applicable.
